# Controllable construction of cobalt nanoparticles in nitrogen-doped carbon nanotubes for photothermal CO_2_ methanation†

**DOI:** 10.1039/d5sc02602d

**Published:** 2025-06-17

**Authors:** Zhanghui Xia, Jianxin Zhai, Longfei Lin, Xiao Chen, Cheng Xue, Shuaiqiang Jia, Jiapeng Jiao, Mengke Dong, Wanying Han, Xinrui Zheng, Teng Xue, Haihong Wu, Buxing Han

**Affiliations:** a Shanghai Key Laboratory of Green Chemistry and Chemical Processes, State Key Laboratory of Petroleum Molecular & Process Engineering, School of Chemistry and Molecular Engineering, East China Normal University Shanghai 200062 China hhwu@chem.ecnu.edu.cn; b Beijing National Laboratory for Molecular Sciences, CAS Laboratory of Colloid and Interface and Thermodynamics, CAS Research/Education Center for Excellence in Molecular Sciences, Center for Carbon Neutral Chemistry, Institute of Chemistry, Chinese Academy of Sciences Beijing 100190 China linlongfei@iccas.ac.cn hanbx@iccas.ac.cn; c Institute of Eco-Chongming Shanghai 202162 China; d School of Chemical Sciences, University of Chinese Academy of Sciences Beijing 100049 China

## Abstract

The development of non-noble metal catalysts for efficient CO_2_ methanation reaction under mild conditions remains a significant challenge. Herein, a non-noble metal catalyst, cobalt nanoparticles (Co NPs) encapsulated within the hollow channels of nitrogen-doped carbon nanotubes (Co@CN-700), was prepared by a pyrolysis-reduction strategy for photothermal CO_2_ methanation. Remarkably, the Co@CN-700 catalyst achieved a prominent CH_4_ production rate of 199.4 mmol g_cat_^−1^ h^−1^ with near-unity selectivity (99.4%) and high CO_2_ conversion (85.8%) at 250 °C, which is outstanding compared to the catalysts reported. The electromagnetic simulation and density functional theory calculations demonstrated that the plasmonic resonance effect of Co NPs enhances the local electric field and thereby alters the intermediate states and rate-limiting step to facilitate CO_2_ methanation. This work offers a straightforward and effective approach for designing non-noble metal catalysts with high activity, selectivity, and stability.

## Abstract 2

This work reports a Co@CN-700 catalyst for photothermal CO_2_ methanation, which could achieve a prominent CH_4_ production rate of 199.4 mmol g_cat_^−1^ h^−1^ with near-unity selectivity (99.4%) and high CO_2_ conversion (85.8%).

## Graphical abstract

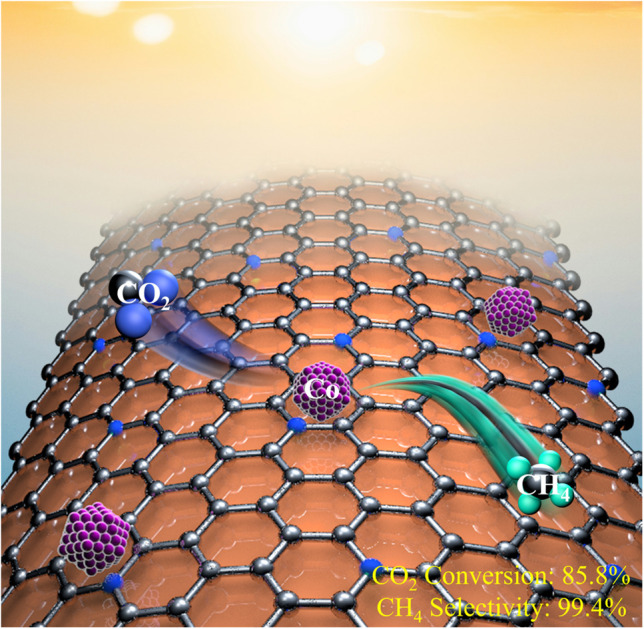

## Introduction

Use of fossil fuels releases a large amount of CO_2_, resulting in environmental and climate problems, and much attention has been paid to develop effective mitigation strategies. A promising approach involves converting CO_2_ with hydrogen into valuable chemicals and fuels such as methane.^1–4^ The produced methane can be readily integrated into existing natural gas pipeline networks, enabling commercial-scale deployment.^5–8^ Additionally, this approach offers a practical solution for hydrogen storage, addressing the technical challenges associated with hydrogen storage, transportation, and large-scale utilization.

CO_2_ methanation, commonly referred as the Sabatier reaction, is an exothermic process that is thermodynamically favored at low temperatures.^9–11^ Over the past decade, various heterogeneous catalysts have been studied to enhance the efficiency of photothermal CO_2_ methanation.^12–17^ Noble metal catalysts have attracted significant attention due to their excellent catalytic performance.^15,18,19^ For instance, Li *et al.* developed a Ru@Ni_2_V_2_O_7_ catalyst that achieved a methanation rate of 114.9 mmol g_cat_^−1^ h^−1^ at 350 °C.^20^ Recently, Zhong *et al.*^15^ reported an Au/Ce_0.95_Ru_0.05_O_2_ solid solution catalyst for the photothermal Sabatier reaction with a methane generation rate of 473 mmol g_cat_^−1^ h^−1^. However, the high cost of noble metals limits their industrial applications, highlighting the need for alternative non-noble metal catalysts with comparable activity and stability. Nickel-based catalysts have shown promise in CO_2_ methanation.^21–23^ By optimizing the size of Ni nanoparticles and tailoring metal–support interactions, a CH_4_ production rate of 63 mmol g^−1^ h^−1^ with a selectivity of 99.8% was achieved.^21^ Cobalt-based catalysts have also been extensively studied for CO_2_ methanation.^24–29^ However, their performance has been hindered by low CH_4_ production rates, typically below 20 mmol g^−1^ h^−1^ (ESI Table S1†), and the formation of undesirable by-products. Improving the activity of non-noble metal catalysts and minimizing by-product generation remain critical challenges for achieving efficient solar-driven Sabatier reactions.

Here, we report a catalyst (Co@CN-700) where carbon nanotubes serve as anchors for uniformly dispersed Co sites, facilitating the catalytic hydrogenation of carbon dioxide to methane with high selectivity. The Co@CN-700 catalyst achieved a very high photothermal CH_4_ production rate of 199.4 mmol g_cat_^−1^ h^−1^, with near-unity selectivity (99.4%), and a CO_2_ conversion of 85.8%. The excellent physical and chemical adsorption of CO_2_ could induce CO_2_ coverage on the catalyst surface and accelerate the methanation reaction. The associated spectral characterization and theoretical calculations showed that the synergistic effect of light energy and heat energy accelerates the conversion of *COOH to *CO species, thereby increasing methanation activity.

## Results and discussion

### Synthesis and characterization of catalysts

**Fig. 1:**
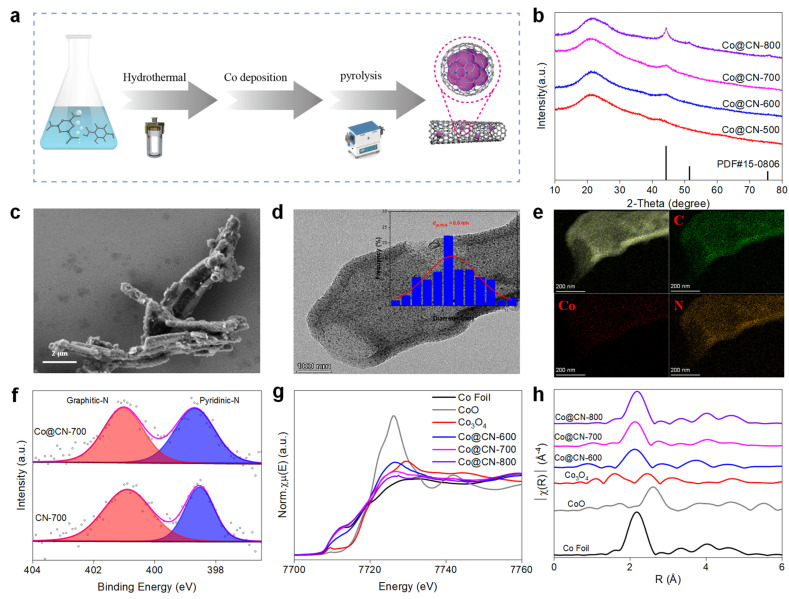
Preparation and characterisation of the catalysts. (a) Schematic diagram of catalyst synthesis and preparation; (b) XRD patterns of various Co-based catalysts; (c) SEM images of the Co@CN-700 sample; (d) TEM images of Co@CN-700 (inset: the average distribution of Co NP size); (e) EDS mapping images of Co@CN-700; (f) high-resolution N 1s XPS spectra of CN-700 and Co@CN-700 catalysts; (g) XANES spectra of Co@CN-600, Co@CN-700, Co@CN-800, and reference samples; (h) EXAFS spectra of Co@CN-600, Co@CN-700, Co@CN-800, and reference samples in *R* space.

The Co@CN-*x* (*x* denotes pyrolysis temperature) catalysts were synthesized by hydrothermal processing, followed by metal deposition and pyrolysis treatment, as outlined in Fig. 1a (detailed synthesis procedures are available in the ESI section†). Inductively coupled plasma optical emission spectrometry (ICP-OES) showed that the Co loading on CN ranged from 6.3% to 11.3% (ESI Table S2†). X-ray diffraction (XRD) patterns revealed no distinct Co metal peaks in the Co@CN-500 sample (Fig. 1b), indicating that Co species are highly dispersed on CN. As pyrolysis temperature increased from 500 to 800 °C, Co metal (PDF# 15-0806) peaks became more pronounced. Besides, Raman spectra (ESI Fig. S1†) showed that Co@CN-700 (*I*_D_/*I*_G_ = 2.02) exhibited fewer defects than CN-700 (*I*_D_/*I*_G_ = 2.41), implying that Co species occupied defective carbon sites. N_2_ adsorption–desorption isotherms (ESI Fig. S2†) show that the Co@CN-*x* catalysts comprised micropores and mesopores, with a Brunauer–Emmett–Teller (BET) surface area of approximately 400 m^2^ g^−1^ (ESI Table S2†), potentially facilitating the diffusion of reactive molecules and exposing active sites. Scanning and transmission electron microscope (SEM/TEM) showed that all Co@CN-*x* and CN samples exhibit hollow nanotube structures with surface wrinkles (Fig. 1c, d and ESI Fig. S3–S6†). The average Co nanoparticle size increased from 5.8 to 8.6 nm and then to 20.7 nm with increasing pyrolysis temperatures from 600 to 800 °C (Fig. 1d and ESI Fig. S7 and S8†). Energy-dispersive X-ray spectroscopy (EDS) mapping confirmed uniform N doping on the graphene support (Fig. 1e), while Co species were highly dispersed within the graphene nanotube pores.

The N 1s XPS spectra showed peaks at 401.0 and 398.6 eV, corresponding to graphitic-N and pyridinic-N,^12^ respectively (Fig. 1f). X-ray absorption fine structure spectroscopy (XAFS) was applied to investigate the local environment of Co species. The X-ray absorption near-edge structure (XANES) results revealed that the intensity and edge energy of the Co@CN-600 catalyst were higher than those of Co foil but lower than those of CoO (Fig. 1g), indicating a partial positive charge. With an increase in reduction temperature from 600 to 700 °C, intensity and the edge energy increased, suggesting that Co species were approaching zero valence. A further increase in reduction temperature to 800 °C did not markedly change the intensity. Extended X-ray absorption fine structure (EXAFS) spectra and fitting data are presented in Fig. 1h and ESI Fig. S9–S14.† In the *R* space, Co–Co scattering (2.49 Å) increased notably with higher reduction temperatures, indicating substantial changes in the coordination environment of Co species.^30^ This result was further corroborated by Co K-edge wavelet transform (WT)-EXAFS analysis (ESI Fig. S15†). The coordination number of Co–Co increased with increasing Co particle size, from 4.2 (Co@CN-600) to 5.5 (Co@CN-700) and 7.7 (Co@CN-800) (details in ESI Table S3†).

### Catalytic performances of Co@CN catalysts for CO_2_ methanation

**Fig. 2:**
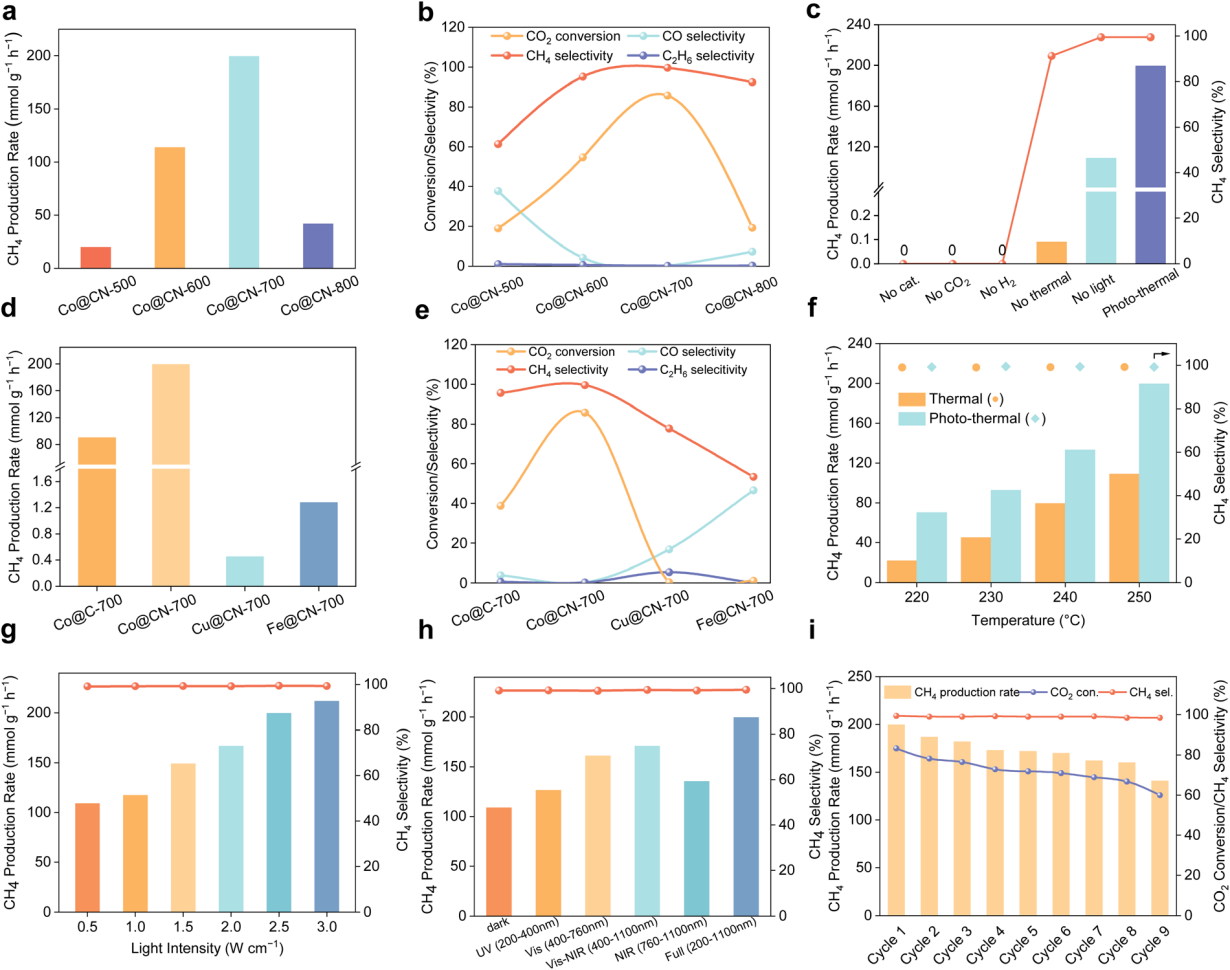
Photothermal catalytic performance. (a) CH_4_ evolution rate over Co@CN-*x* catalysts; (b) product distribution over Co@CN-*x* catalysts; (c) control experiments for the Co@CN-700 sample under different conditions. Reaction conditions: 15 mg catalyst, full-arc 300 W UV–xenon lamp, 2.5 W cm^−2^, 250 °C, irradiation time 4 hours, initial pressure 1 MPa (H_2_/CO_2_ = 4/1). (d) CH_4_ evolution rate over different catalysts; (e) product distribution over different catalysts; (f) temperature-dependent CH_4_ generation rate over Co@CN-700 under photothermal and thermal conditions; (g) influence of light intensity on the CH_4_ evolution rate over Co@CN-700; (h) the impact of various light wavelengths on the catalytic performance of the Co@CN-700 catalyst; (i) catalytic stability tests of Co@CN-700 under photothermal conditions. Reaction conditions: 15 mg of catalyst, full-arc 300 W UV–xenon lamp, 2.5 W cm^−2^, 250 °C, irradiation time 4 h, initial pressure 1 MPa (H_2_/CO_2_ = 4/1).

The CO_2_ methanation performance of Co@CN catalysts was evaluated in a photothermal reactor with an external heating system. The reaction temperature was controlled by external heating in combination with irradiation from an Xe lamp (ESI Fig. S16 and S17†). Reaction parameters, including the H_2_/CO_2_ ratio, pressure, and irradiation time, were optimized for CO_2_ methanation (ESI Fig. S18–S20†). The optimized conditions (H_2_/CO_2_ ratio of 4, a pressure of 1 MPa, and an irradiation time of 4 h) were then applied for the catalytic studies. CO_2_ methanation activity was tested across Co@CN-*x* catalysts prepared at different pyrolysis/reduction temperatures (Fig. 2a and b). The catalytic activity displayed a volcano-like trend in relation to the pyrolysis/reduction temperature. Notably, Co@CN-700 achieved a CH_4_ production rate of 199.4 mmol g_cat_^−1^ h^−1^ with nearly 100% selectivity, setting a new benchmark in photothermal CO_2_ methanation activity for non-noble metal catalysts (ESI Table S1†). Moreover, no liquid products were detected in ^1^H NMR spectroscopy (ESI Fig. S21†).

Controlled catalytic experiments in the absence of CO_2_ or H_2_ revealed that no CH_4_ or other carbonaceous products were detected (Fig. 2c), which proved that the products originated from CO_2_ during the reaction. Tests with no catalyst, no thermal input, or no light showed either no or reduced activity compared to the photothermal catalysis on Co@CN-700 (Fig. 2c). These indicate that both the catalysts and photothermal treatments are essential for achieving high CO_2_ methanation activity and selectivity. The results (ESI Fig. S22†) showed that the CH_4_ production rates were 1.28, 0.26 and 0.05 mmol g^−1^ h^−1^ over the Co-30, Co-50 and Co-100 samples, which were much lower than the catalytic activity of Co@CN-700. This shows that the size of Co NPs and the support have important roles in the catalytic performance. CH_4_ production rates of 90.1 and 199.4 mmol g_cat_^−1^ h^−1^ were observed for Co@C-700 and Co@CN-700 catalysts, respectively (Fig. 2d). CH_4_ selectivity increased from 23.5% for Co-30 to 95.7% for Co@C-700 and reached 99.6% for Co@CN-700 (Fig. 2e). Furthermore, Cu@CN-700 and Fe@CN-700 exhibited negligible photothermal catalytic activity, underscoring the superior catalytic performance of Co in this study.

Compared to pure thermo-catalysis, the CH_4_ production rate increased significantly when photon energy was coupled with external thermal energy (Fig. 2f), suggesting that photon energy injection effectively enhances CO_2_ methanation. The relationship between photothermal methanation activity and light intensity was investigated on Co@CN-700, indicating a substantial increase in the CO_2_ methanation rate at higher light intensity (Fig. 2g). To evaluate the contribution of different wavelengths, CO_2_ methanation was conducted under UV, visible (Vis), near-infrared (NIR), and Vis-NIR irradiation (Fig. 2h). The CH_4_ production rate increased by 16% under UV light (200–400 nm, 126.4 mmol g_cat_^−1^ h^−1^) compared to the dark conditions (108.9 mmol g_cat_^−1^ h^−1^) and by 24% under NIR light (760–1100 nm, 135.3 mmol g_cat_^−1^ h^−1^). Notably, vis light (400–760 nm) led to a 48% increase in the CH_4_ production rate (161 mmol g_cat_^−1^ h^−1^) over dark conditions, suggesting that the photon energy of visible light plays a dominant role in the photothermal process. These findings demonstrated that the light of different wavelengths displays promotion in CO_2_ methanation, emphasizing the critical role of photon energy strength in the photothermal reactions.

The stability of Co@CN-700 was studied by cycling tests, in which the CH_4_ production rate exceeded 160 mmol g_cat_^−1^ h^−1^ and CH_4_ selectivity was maintained above 99% over nine cycles (Fig. 2i). In order to investigate the slight deactivation of the catalyst, XPS, SEM and TEM were performed. The XPS results (ESI Fig. S25†) showed that the valence states of Co, C and N in the catalyst did not change obviously compared with the fresh catalyst. The SEM results (ESI Fig. S24†) demonstrated that the morphology of the catalyst remained unchanged after utilization. The above results show that the morphology and valence state of the catalyst were not the cause of deactivation. However, the TEM results (ESI Fig. S26†) demonstrated that, in comparison with the particle size of the fresh catalyst (8.6 nm), the particle size of Co increased to 13.2 nm after nine cycles. This finding suggests that the gradual increase in particle size may be the primary factor contributing to the slow deactivation of the catalyst.

### Roles of active sites and support of Co@CN catalysts

**Fig. 3:**
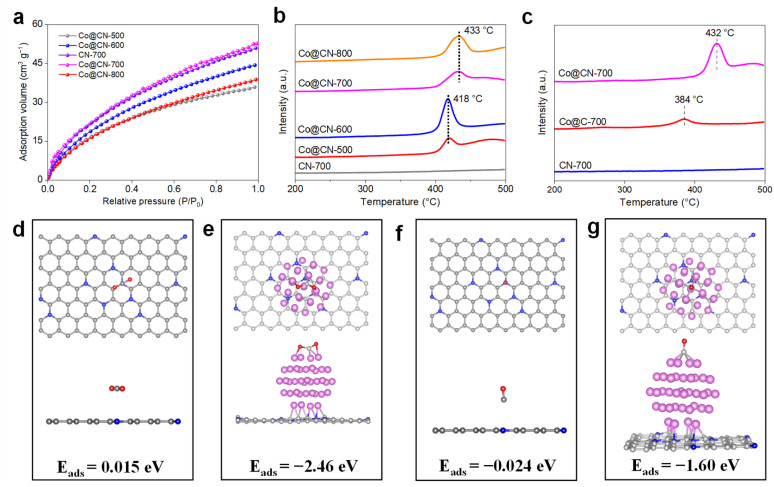
Physical and chemical adsorption of CO_2_ and CO on catalysts. (a) CO_2_ adsorption on various Co-based catalysts; (b) CO_2_-TPD profiles of various Co-based catalysts; (c) CO-TPD profiles of various Co-based catalysts; optimized configuration for CO_2_ adsorption on the CN substrate (d) and Co@CN surface (e); optimized configuration for CO adsorption on the CN substrate (f) and Co@CN surface (g).

To elucidate the adsorption properties of Co@CN catalysts, CO_2_ physical adsorption and CO_2_ temperature-programmed desorption (CO_2_-TPD) experiments were conducted. Fig. 3a illustrates that the Co@CN-700 sample exhibited the highest CO_2_ physical adsorption capacity, with the CN-700 sample showing only a slight decrease in this capacity. This result suggests that CO_2_ physical adsorption capacity mainly originated from the porous, hollow nanotube structures with surface wrinkles. Interestingly, the CO_2_ physical adsorption capacity followed the order Co@CN-700 > Co@CN-600 > Co@CN-800 > Co@CN-500, which corresponds directly to the observed methane production capacity.

The CO_2_-TPD profiles in Fig. 3b display a pronounced peak between 400 and 450 °C for all Co@CN-X catalysts, while the CN-700 sample displayed no detectable CO_2_ desorption peaks, indicating that efficient CO_2_ chemical adsorption occurs specifically on Co sites. Furthermore, density functional theory (DFT) calculations were performed to study adsorption behavior of CO_2_ on various surfaces. The calculated adsorption energies of CO_2_ on the nitrogen-doped carbon substrates and Co@CN-700 are 0.015 eV and −2.46 eV, respectively (Fig. 3d and e), confirming that Co sites significantly enhance CO_2_ adsorption, consistent with the CO_2_-TPD results.

Given that CO is an intermediate in the CO_2_ methanation, it is crucial to investigate adsorption behavior of CO on different catalysts. The CO-TPD profile (Fig. 3c) for the Co@CN-700 sample shows a strong CO desorption peak at 432 °C, while the CN-700 sample exhibited no CO desorption peaks, suggesting that efficient CO chemical adsorption occurs on Co sites. DFT calculations revealed that the adsorption energies of CO on the nitrogen-doped carbon substrate and Co@CN-700 catalyst were −0.024 eV and −1.60 eV, respectively (Fig. 3f and g). The above results demonstrate that the N-doped graphene nanotube structure enhances the local concentration of CO_2_ near the catalyst *via* physical adsorption, followed by further capture through chemical adsorption at the Co sites. Concurrently, H_2_ is efficiently activated at these Co sites,^31,32^ initiating the hydrogenation process by reacting with the adsorbed CO_2_ on the surface. Moreover, the Co@CN-700 catalyst exhibits substantially improved CO adsorption capacity, which retards CO desorption from the catalyst surface and improves methane selectivity.

### Light and thermal properties of Co@CN catalysts

**Fig. 4:**
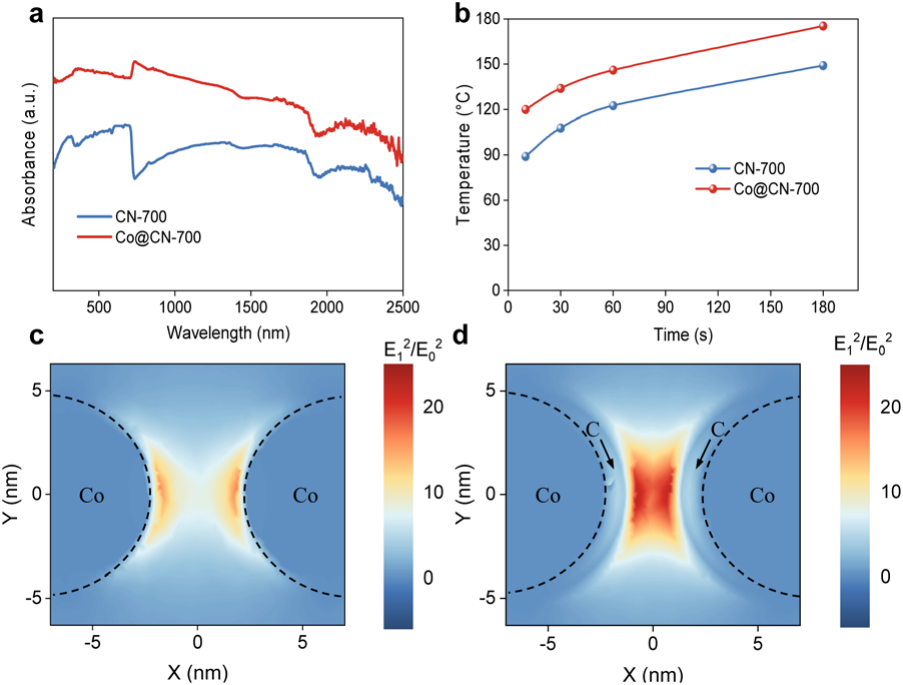
Light and thermal properties of catalysts. (a) UV–vis–IR absorption spectra of the CN-700 and Co@CN-700 samples. (b) Surface temperature of different samples collected using an infrared thermometer. Spatial distribution of electric field intensity induced by the localized surface plasmon resonance, from FDTD simulation of (c) 8.6 nm Co NPs, and (d) composite of Co NPs (8.6 nm) and a carbon layer (1 nm).

The efficient utilization of light is a critical factor that influences photothermal catalytic activity. To study light absorption capacity, the UV–Vis–NIR absorption spectra of various samples were measured (Fig. 4a). The CN-700 sample demonstrated a broad absorption peak from 250 to 2500 nm, implying strong broad spectrum light adsorption. The Co@CN-700 sample exhibited even stronger light absorption across the entire solar spectral range, attributed to the localized surface plasmon resonance (LSPR) effect of Co metal.^33^ This result indicates that metal-carbon composites enhance the light absorption properties of the catalysts *via* the plasmon–photon coupling. In addition, Co@CN-700 displays a more pronounced photothermal effect than CN-700 when irradiated at a power density of 2.5 W cm^−2^ (Fig. 4b and ESI Fig. S27†).^34,35^ A three-dimensional finite difference time domain (FDTD) simulation was conducted to simulate the local electric field distribution in the catalysts. Under single-wavelength light (700 nm), the electric field distribution appeared uneven, with peak intensities on the surfaces of metal nanoparticles and an exponential decrease with distance from the surface (Fig. 4d). However, the local electric field intensity induced by LSPR significantly increased upon the incorporation of Co nanoparticles and a carbon layer, indicating an enhancement in hot electron production capabilities. These hot electrons can transfer to the adsorbed reactant molecules, thereby facilitating their activation at the nanoparticle–molecule interface.^36–38^ This process increases the probability of electronic or vibrational transitions in the adsorbed molecules, ultimately accelerating the chemical reaction.^33^

### Reaction mechanism

**Fig. 5:**
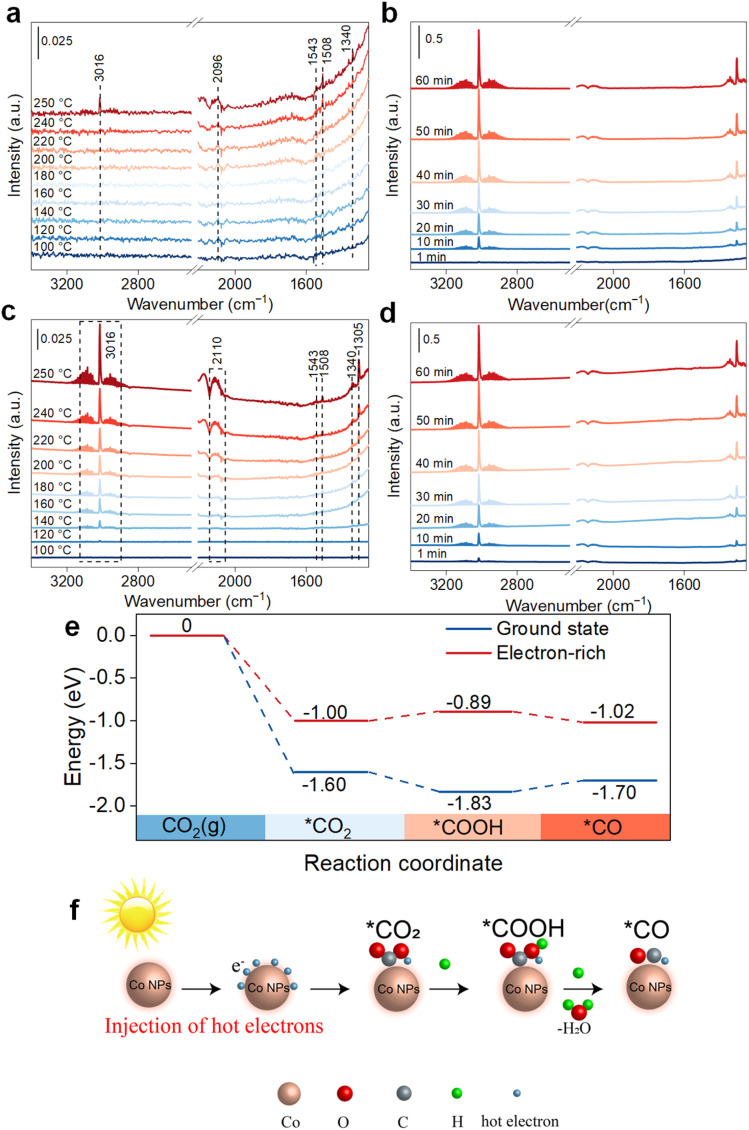
Proposed reaction mechanism. *In situ* FTIR spectra of thermal (a and b) and photothermal (c and d) methanation reactions over Co@CN-700. Reaction conditions: full-spectrum light irradiation, a H_2_ and CO_2_ (50/50 vol%) atmosphere; (e) free energy of Co@CN-700 at the ground state and electron-rich state, respectively; (f) proposed photothermal catalytic reaction mechanism for CO_2_ hydrogenation.

To get insight into the reaction mechanism of CO_2_ methanation under photothermal conditions, *in situ* Fourier-transform infrared spectroscopy (FTIR) was implemented to detect surface chemical intermediates. Under thermal catalysis (Fig. 5a and b), vibration peaks at 1508 (m-CO_3_^2−^), 1543 (COOH*), and 2096 cm^−1^ (*CO)^21,35^ became more prominent as the temperature increased from 100 °C to 250 °C. Notably, a peak at 3016 cm^−1^, corresponding to CH_4_ appeared at 250 °C and intensified over time (Fig. 5b). In photothermal catalysis, the FTIR results showed similar peaks for COOH*, m-CO_3_^2−^, and the *CO intermediate (Fig. 5c and d). However, the bands at 1305 (bending vibration of C–H) and 3016 cm^−1^ (stretching vibration of C–H)^39^ assigned to CH_4_ emerged at 140 °C, implying that CO_2_ methanation can proceed at lower temperatures under photothermal conditions. Additionally, the intensities of intermediate peaks in photothermal catalysis were consistently higher than those observed in pure thermal catalysis.

First-principles density functional theory (DFT) calculations^40,41^ were conducted for both the ground and the electron-rich states (Fig. 5e). In the ground state, the rate-limiting step is the conversion of *COOH to *CO, with a barrier energy of 0.13 eV. In the electron-rich state, the conversion of *COOH to *CO becomes more favorable, while the rate-limiting step shifts to the conversion of *CO_2_ to *COOH, which requires a lower barrier energy of 0.11 eV. This phenomenon can be attributed to the direct involvement of hot electrons in the reaction, altering the intermediate states and facilitating subsequent steps (Fig. 5f). Consequently, the intensified local electric field, induced by anchoring of Co nanoparticles on carbon nanotubes, enhanced CO_2_ methanation by promoting the conversion of *COOH to *CO.

## Conclusions

In summary, a collaborative photothermal catalytic strategy was developed to enhance the efficiency of CO_2_ hydrogenation to CH_4_. The Co@CN-700 catalyst achieved record-breaking performance, with a CH_4_ production rate of 199.4 mmol g_cat_^−1^ h^−1^ and a selectivity of 99.4% at 250 °C. The LSPR effect of Co nanoparticles amplified the local electric field, enhancing the photothermal effect in synergy with thermal energy to change the rate-limiting step and lower the barrier energy of reactions. This study provides valuable insights for designing efficient and stable non-noble metal catalysts for photothermal CO_2_ methanation.

## Author contributions

Conceptualization: ZX, JZ and XC. Methodology: ZX and LL. Investigation: ZX, CX, SJ and MD. Visualization: ZX, SJ, JJ and MD. Supervision: ZX, WH, XZ and TH. Writing—original draft: ZX and LL. Writing – review & editing: ZX, LL, HW and BH.

## Conflicts of interest

The authors declare no competing interests.

## Supplementary Material

SC-OLF-D5SC02602D-s001

## Data Availability

All data are available in the main text or the ESI.†
