# Supplementary material for: Controllable construction of cobalt nanoparticles in nitrogen-doped carbon nanotubes for photothermal CO2 methanation
Source: Chem Sci. 2025 Jun 17;16(29):13382–9. doi: 10.1039/d5sc02602d (PMC12188655; doi:10.1039/d5sc02602d)
Supplement: SC-016-D5SC02602D-s001 [file SC-016-D5SC02602D-s001.pdf]

Supplementary Information (SI) for Chemical Science.
This journal is © The Royal Society of Chemistry 2025

Supporting Information

Controllable Construction of Cobalt Nanoparticles in Nitrogen-Doped Carbon Nanotubes for Photothermal CO_2 Methanation

Zhanghui Xia[1,3], Jianxin Zhai[1,3], Longfei Lin[2,4,*], Xiao Chen[1,3], Cheng Xue[1,3], Shuaiqiang Jia[1,3], Jiapeng Jiao[1,3], Mengke Dong[1,3], Wanying Han[1,3], Xinrui Zheng[1,3], Teng Xue[1,3], Haihong Wu[1,3,*], and Buxing Han[1,3,4,*]

1 Shanghai Key Laboratory of Green Chemistry and Chemical Processes, State Key Laboratory of Petroleum Molecular & Process Engineering, School of Chemistry and Molecular Engineering, East China Normal University, Shanghai, 200062, China

2 Beijing National Laboratory for Molecular Sciences, CAS Laboratory of Colloid and Interface and Thermodynamics, CAS Research/Education Center for Excellence in Molecular Sciences, Center for Carbon Neutral Chemistry, Institute of Chemistry, Chinese Academy of Sciences, Beijing 100190, China.

3 Institute of Eco-Chongming, Shanghai 202162, China

4 School of Chemical Sciences, University of Chinese Academy of Sciences, Beijing 100049, China.

* To whom correspondence should be addressed. Email: linlongfei@iccas.ac.cn (L.L.); hhwu@chem.ecnu.edu.cn (H.W.); hanbx@iccas.ac.cn (B.H.)

Section 1. Materials and characterization

Chemicals

$Co(NO_3)_2{\cdot}6H_2O$ (99%) was purchased from Thermo Fisher Scientific (China). 2,4-dihydroxybenzoic acid (98%) was obtained from Shanghai Macklin Biochemical Co., Ltd. Hexamethylenetetramine (CP), Ethanol (AR), $NH_3{\cdot}H_2O$ (AR), $Cu(NO_3)_2{\cdot}3H_2O$ (AR), and $Fe(NO_3)_2{\cdot}9H_2O$ (AR) were provided by Sinopharm Chemical Reagent Co., Ltd. Pluronic F127 was supplied by Sigma-aldrich. Melamine was purchased from Aladdin Chemistry Co., Ltd. All commercial chemicals were used without any further purification. CO_2 (99.995%) and H_2 (99.9995%) were provided by Air Liquid Houlding Co., Ltd. (China).

Catalysts synthesis

Preparation of Co NPs

The metal Co NPs with different sizes (30, 50 and 100 nm) were purchased from Adamas. These Co NPs catalysts are named Co-30, Co-50 and Co-100, respectively. An appropriate amount of Co NPs was soaked in 0.5 M sulfuric acid under ultrasonic for 0.5 hours before each use.

Preparation of Co@C-700 catalyst

Typically, certain amounts of 2,4-dihydroxybenzoic acid (2.3 g), Hexamethylenetetramine (0.7 g), and F127 (3.0 g) were dissolved in 60 mL deionized water under slow stirring. After stirring for 30 min, the mixed solution was transferred into a 100 mL Teflon-lined stainless-steel autoclave, which was then sealed, heated to 130 °C, and maintained at that temperature for 4 h. Afterwards, the autoclave was cooled to room temperature naturally. The products were collected by filtration, washed three times with deionized water, and finally dried at 80 °C under vacuum for 8 h to obtain the brown-yellow solid powder. Then, 1.0 g of the brown-yellow solid powder and 0.24 g of $Co(NO_3)_2{\cdot}6H_2O$ were dispersed in 48 mL H_2O under vigorous stirring. Following this, 12 mL $NH_3{\cdot}H_2O$ as a precipitant was added dropwise to the above solution and heated at 60 °C for 12 h. After that, the product was washed with deionized water, collected by filtration, and dried at 80 °C in the vacuum oven overnight. 0.2 g of the above Co@C precursor was dispersed in a ceramic boat and put in a tube furnace. The furnace was heated to 700 °C with a ramp rate of 5 °C

min^{-1} and held at this temperature for one hour under a continuous flow of 10 vol% H_2/Ar. After cooling to room temperature naturally, the Co@C-700 sample was obtained.

Preparation of Co@CN Precursor

Typically, certain amounts of 2,4-dihydroxybenzoic acid (2.3 g), Hexamethylenetetramine (0.7 g), melamine (1.0 g), and F127 (3.0 g) were dissolved in 60 mL deionized water under slow stirring. After stirring for 30 min, the mixed solution was transferred into a 100 mL Teflon-lined stainless-steel autoclave. The sealed autoclave was heated to 130 °C, and maintained at that temperature for 4 h. Afterwards, the autoclave was cooled to room temperature naturally. The products were collected by filtration, washed three times with deionized water, and finally dried at 80 °C under vacuum for 8 h to obtain the brown-yellow solid powder. Then, 1.0 g of the brown-yellow solid powder and 0.24 g of $Co(NO_3)_2\cdot 6H_2O$ were dispersed in 48 mL H_2O under vigorous stirring. Following this, 12 mL $NH_3\cdot H_2O$ as a precipitant was added dropwise to the above solution and heated at 60 °C for 12 h. After that, the product was washed with deionized water, collected by filtration, and dried at 80 °C in the vacuum oven overnight. Finally, the Co@CN precursor was obtained.

Preparation of Co@CN catalysts

In a typical preparation of Co@CN-700, 0.2 g of the above Co@CN precursor was dispersed in a ceramic boat and put in a tube furnace. The furnace was heated to 700 °C with a ramp rate of 5 °C min^{-1} and held at this temperature for one hour under a continuous flow of 10 vol% H_2/Ar. After cooling to room temperature naturally, the Co@NC-700 sample was obtained. The other catalysts were prepared using the aforementioned procedure and denoted as Co@NC-x (x stands for pyrolysis temperature).

Photothermal CO_2 hydrogenation

Photothermal CO_2 hydrogenation was performed in a stainless steel reactor with a total volume of 180 mL (CEL-MPR, Beijing China Education Au-Light Co., Ltd.). In a typical run, 0.015 g of the catalyst powder was dispersed in 5 mL water under ultrasound conditions. The above catalyst dispersion was evenly dripped onto the reactor with an illuminated area of 12.6 cm^2 and heated in the vacuum oven (333K) overnight to volatilize the solvent to obtain the catalyst film. Prior to the photothermal

reaction for every experiment, the reactor was filled and evacuated with CO_2 three times to remove the air. Then the mixed gas of CO_2/H_2 (1MPa) was charged into the reactor at room temperature. After that, the reactor system was heated up to 250°C through the combined contribution of external heating and the 300W UV-Xe lamp (Beijing China Education Au-Light Co., Ltd). The light intensity on the catalyst surface was measured to be 2.5 Wcm^{-2} by an optical power meter (Beijing China Education Au-Light Co., Ltd). After a reaction for 4h, the gas products were analyzed by gas chromatograph (GC, 8860B, Agilent) equipped with a thermal conductor detector (TCD) and flame ionization detector (FID).

Subsequent to the conclusion of the reaction, the catalyst film is returned to the vacuum oven for desiccation, and the reaction kettle is subjected to a further cycle of cleaning and drying. The dry catalyst film was then introduced into the reactor, where the photothermal CO_2 hydrogenation process was repeated. The aforementioned operation was repeated in order to conduct the stability experiment.

Materials characterization

The Co contents were quantified by an inductively coupled plasma emission spectrometer (ICP-OES) on an Optima8300. Scanning electron microscope (SEM) images were obtained by using a ZEISS Sigma 300. Transmission electron micrography (TEM) images were acquired on an FEI Talos F200x electronic microscopy. UV–VIS–NIR diffuse reflectance spectra were obtained by a UV–VIS–NIR spectrophotometer (UV-3600i Plus, Shimadzu, Japan). XPS measurements were operated on AXIS Supra electron spectrometer. Raman analysis was conducted on a Renishaw inVia Reflex Raman Spectrometer with a 532nm laser. The XRD patterns were acquired on Rigaku Ultima VI. The in situ IR measurements were performed on a Nicolet NEXUS-FTIR-670 spectrometer equipped with a high-temperature cell, operating at a resolution of 4 cm^{-1} and 32 scans per spectrum. The adsorption isotherms of CO_2 were determined at 293 K on a BELSORP-max II equipment. The CO_2-TPD and CO-TPD experiments were carried out on Micromeritics Auto Chem II chemisorption analyser with a TCD detector. The XAS spectra at the Co K-edges were

recorded at the BL11B beamline of Shanghai Synchrotron Radiation Facility (SSRF).

Electromagnetic field simulation. The electric field was calculated by the finite-difference time-domain (FDTD) using COMSOL Multiphysics 6.2. The electric field enhancement distribution near the particle was obtained by adding the physical field of the electromagnetic wave, "frequency domain" and "Wavelength domain". The dielectric functions of Co and graphene were taken from the literature. In the calculation process, the excitation electromagnetic field propagates along the Z axis and oscillates along the X axis, and perfect matching layer (PML) conditions were used in Z directions.

DFT calculations.

The XRD results indicate that mainly Co (111) crystal faces are exposed in the Co@CN-700. Based on this, the Co@CN-700 model is constructed as Co (111) surface of the nanoclusters. Spin-polarized electronic structure calculations were performed using the plane-wave basis set approach as implemented in the Vienna ab initio simulation package (VASP)[1]. The projector augmented wave (PAW) method was used to represent the ion–core electron interactions[2]. The valence electrons were represented with a plane wave basis set with an energy cutoff of 450 eV. Electronic exchange and correlation were described with the Perdew–Burke–Ernzerhof (PBE) functional[3]. DFT-D3 method was used to treat the van der Waals interaction [4]. A 2×2×1 Monkhorst–Pack scheme was used to generate the k-point grid for the modeled surfaces[5]. The convergence criteria for the self-consistent electronic structure and geometry were set to 10^{-5} eV and 0.05 eV/Å, respectively.

Section 2. Figures and Tables

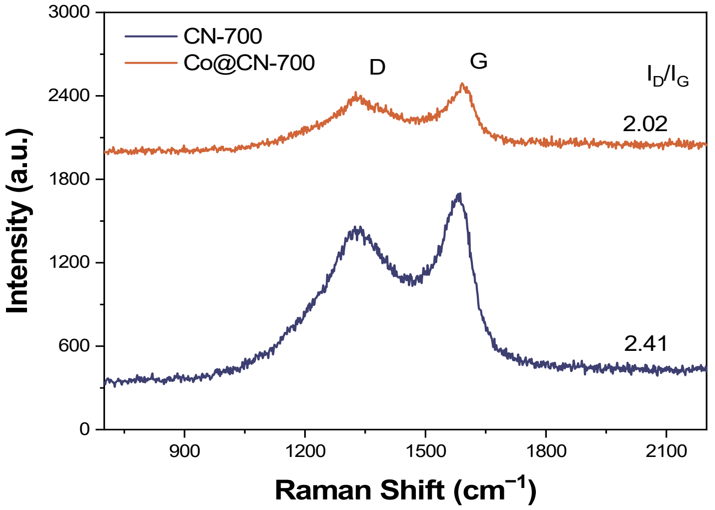

Supplementary Fig.1. Raman spectrum of different catalysts.

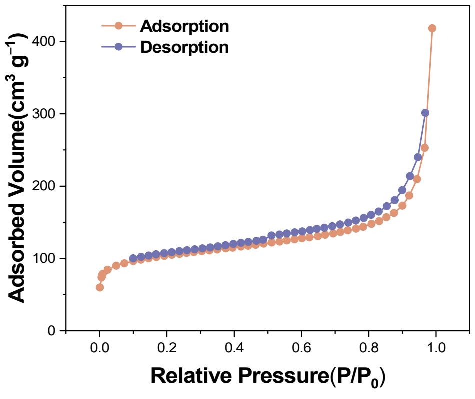

(a)

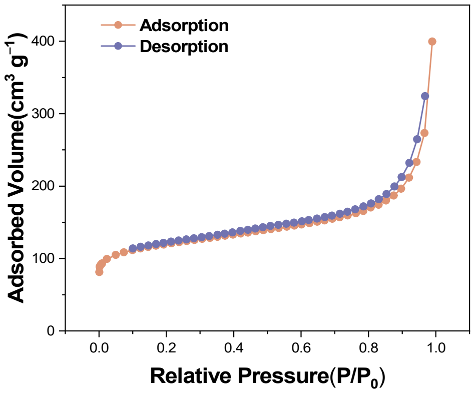

(b)

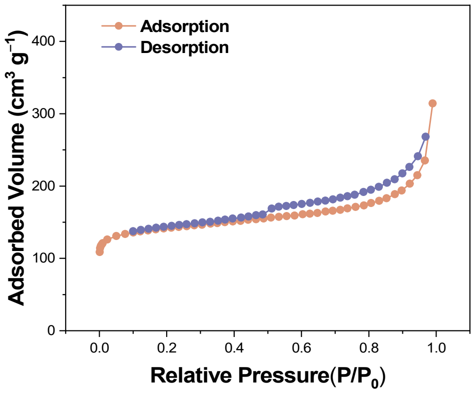

(c)

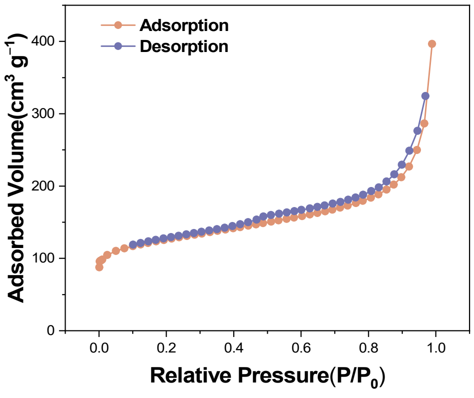

(d)

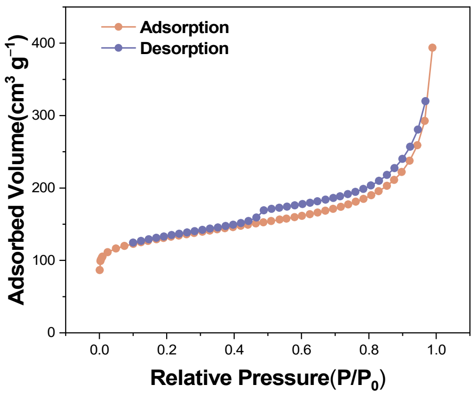

(e)

Supplementary Fig.2. N_2 adsorption–desorption isotherms of the as-prepared Co@CN-500 (a), Co@CN-600 (b), CN-700 (c), Co@CN-700 (d), and Co@CN-800 (e) samples.

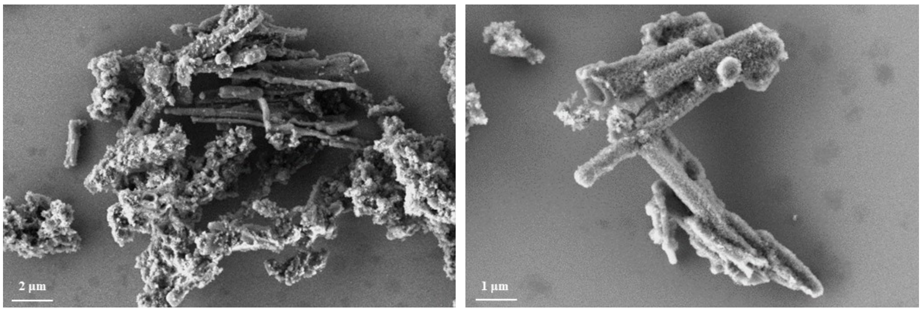

Supplementary Fig.3. SEM images of the Co@CN-500 sample.

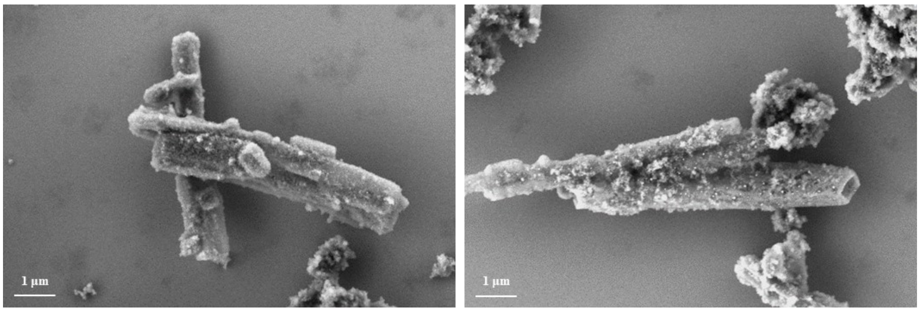

Supplementary Fig.4. SEM images of the Co@CN-600 sample.

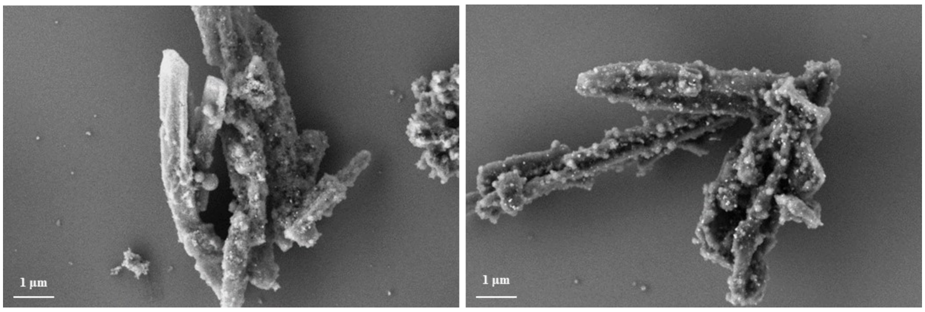

Supplementary Fig.5. SEM images of the Co@CN-800 sample.

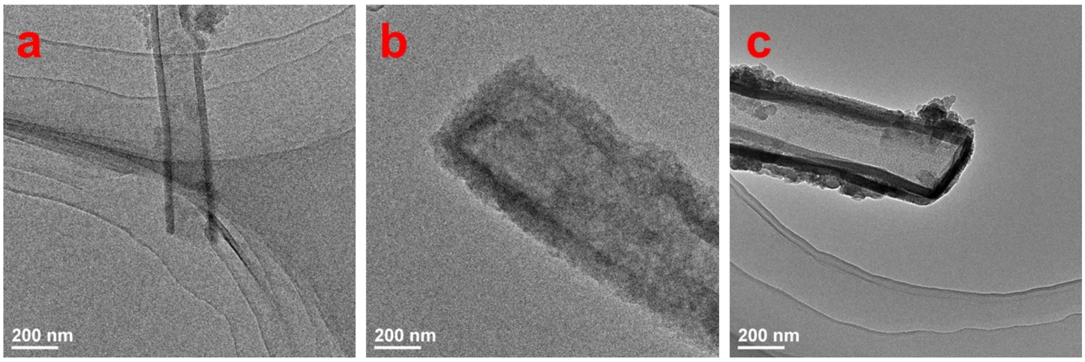

Supplementary Fig.6. TEM images of the CN-800 sample.

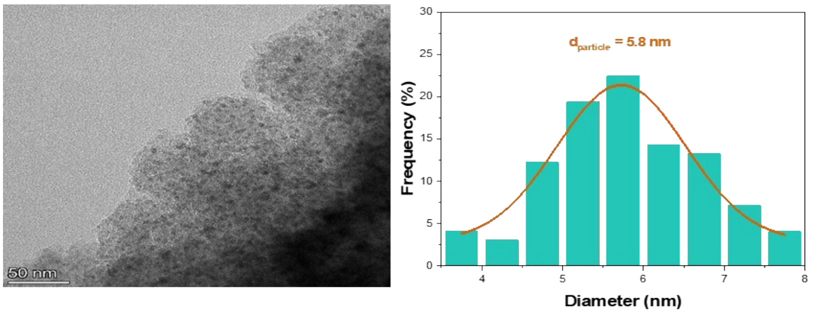

Supplementary Fig.7. The average distribution of Co NPs size of Co@CN-600 sample.

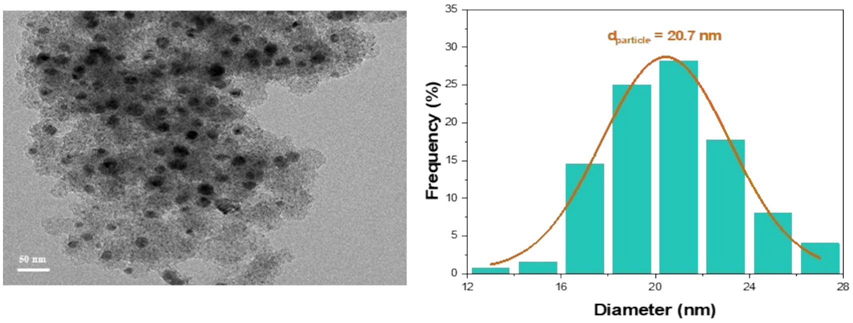

Supplementary Fig.8. The average distribution of Co NPs size of Co@CN-800 sample.

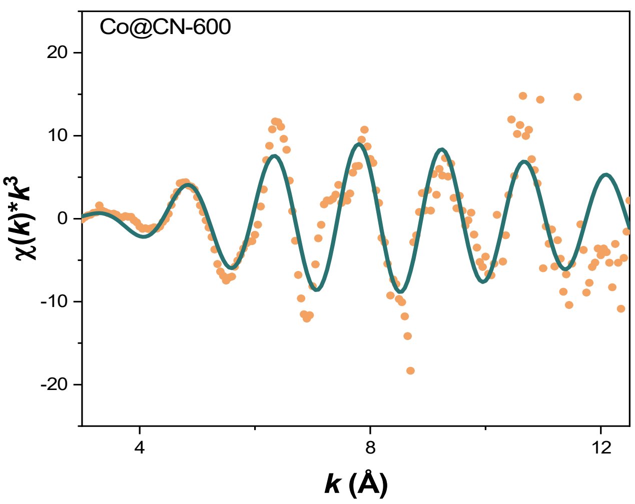

Supplementary Fig.9. Co K-edge EXAFS (points) and the curvefit (line) for Co@CN-600, shown in k^3-weighted k-space.

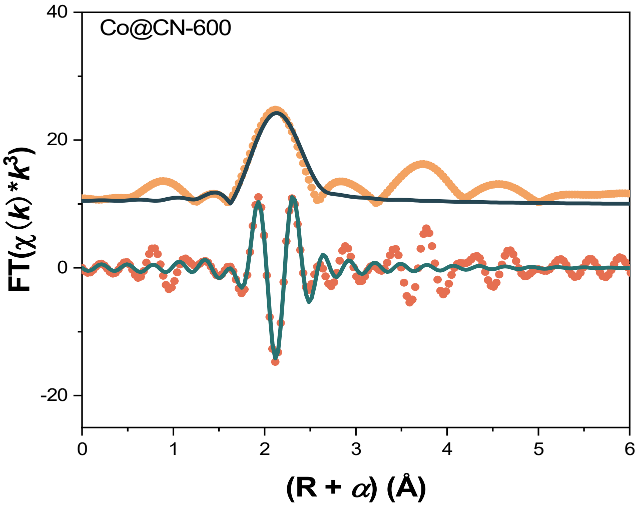

Supplementary Fig.10. Co K-edge EXAFS (points) and curvefit (line) for Co@CN-600, shown in *R*-space (FT magnitude and imaginary component). The data are k^3-weighted and not phase-corrected.

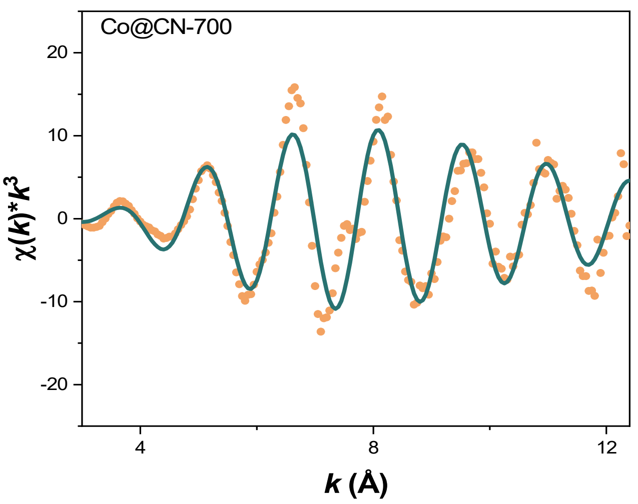

Supplementary Fig.11. Co K-edge EXAFS (points) and the curvefit (line) for Co@CN-700, shown in k^3-weighted k-space.

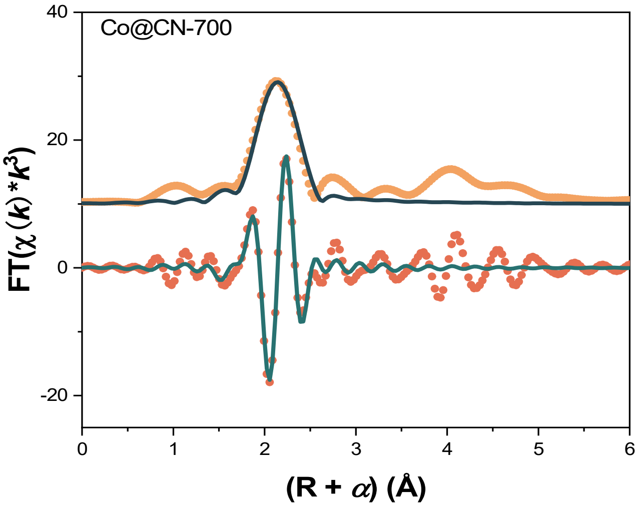

Supplementary Fig.12. Co K-edge EXAFS (points) and curvefit (line) for Co@CN-700, shown in *R*-space (FT magnitude and imaginary component). The data are k^3-weighted and not phase-corrected.

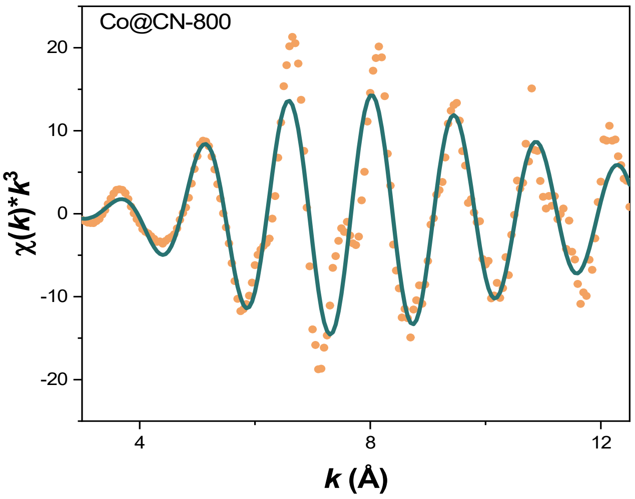

Supplementary Fig.13. Co K-edge EXAFS (points) and the curvefit (line) for Co@CN-800, shown in k^3-weighted k-space.

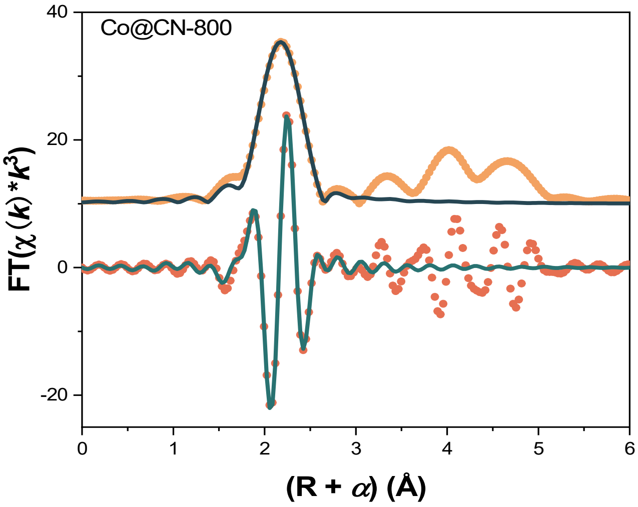

Supplementary Fig.14. Co K-edge EXAFS (points) and curvefit (line) for Co@CN-800, shown in *R*-space (FT magnitude and imaginary component). The data are k^3-weighted and not phase-corrected.

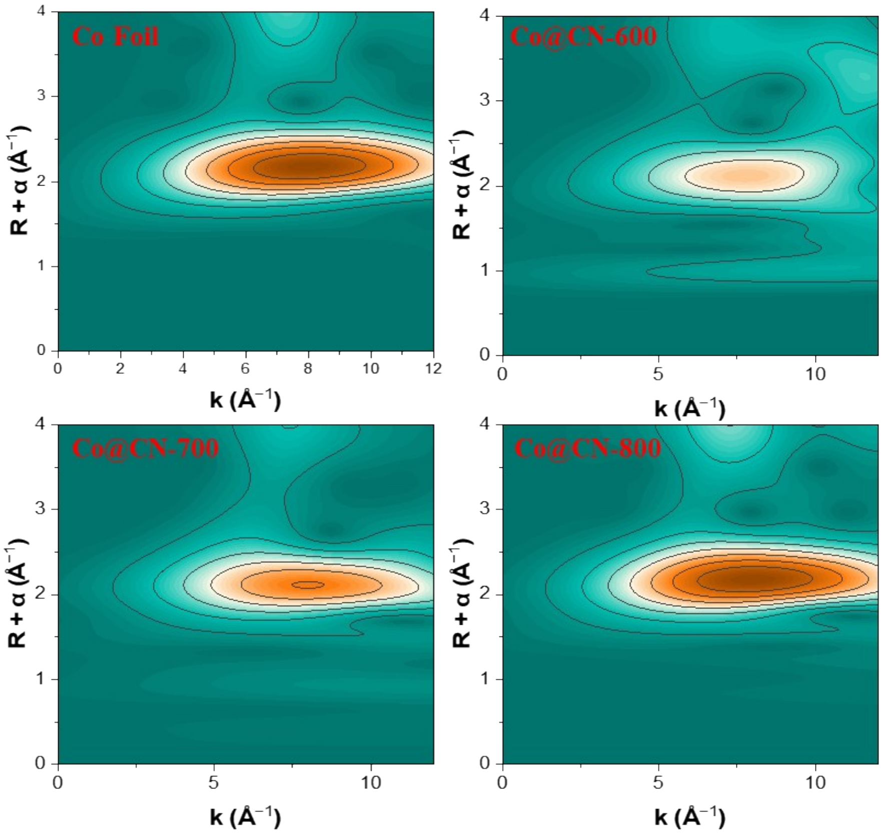

Supplementary Fig.15. Co K-edge wavelet transform (WT)-EXAFS analysis of different catalysts.

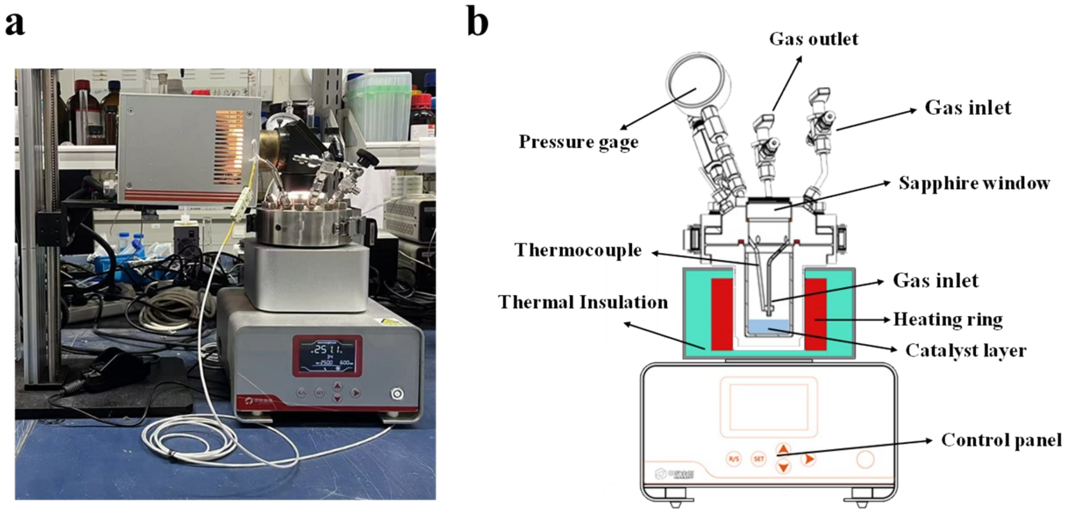

Supplementary Fig.16. (**a**) Photograph of the apparatus setup for photothermal CO_2 experiments in the batch reactor; (**b**) Schematic illustration of the photothermal reactor.

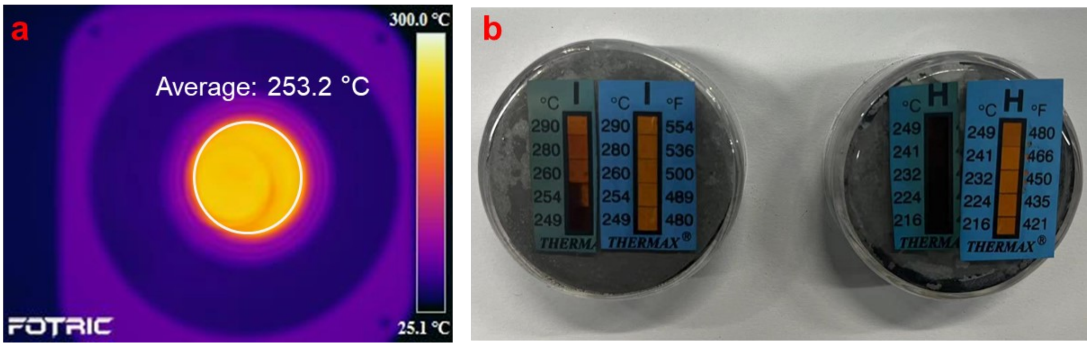

Supplementary Fig.17. (**a**) The catalyst surface temperature on the Co@CN-700 sample under 2.5W cm^{-2} irradiation and external heating (Set temperature: 250 °C) recorded by a Fotric thermal IR imager (Fotric, Shanghai, China); (**b**) Experimental operation of measuring temperature at the bottom of the catalyst with the thermochromic temperature indicators (Thermax, UK).

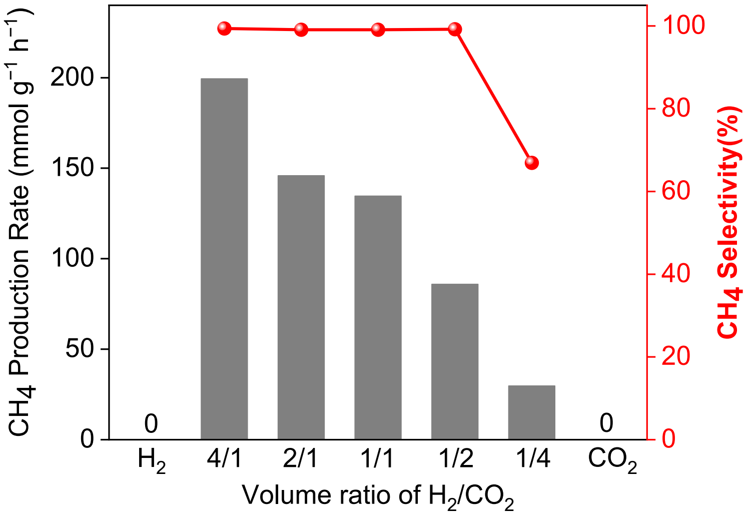

Supplementary Fig.18. Influence of H_2/CO_2 volume ratio in the feedstock on CH_4 evolution rate over Co@CN-700.

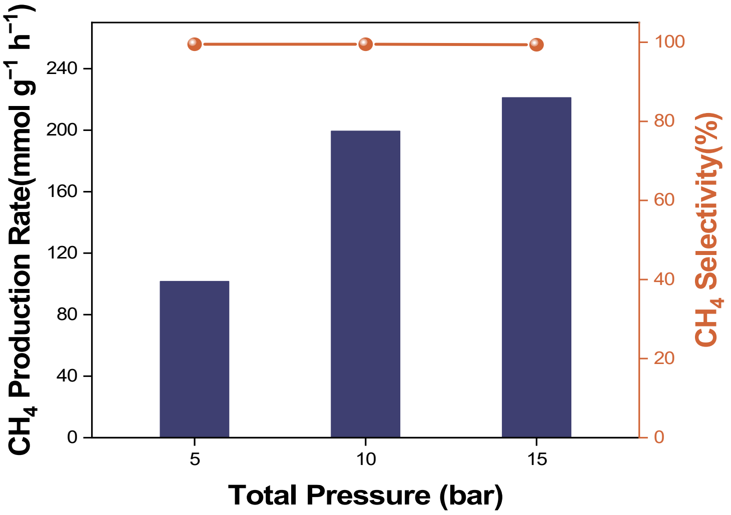

Supplementary Fig.19. Influence of total pressure on CH_4 production rate over Co@NC-700 catalyst. Reaction conditions: 250 °C, 4h, H_2/CO_2=4/1, 15 mg catalyst, full-arc 300 W UV-xenon lamp, 2.5 W cm^{-2}.

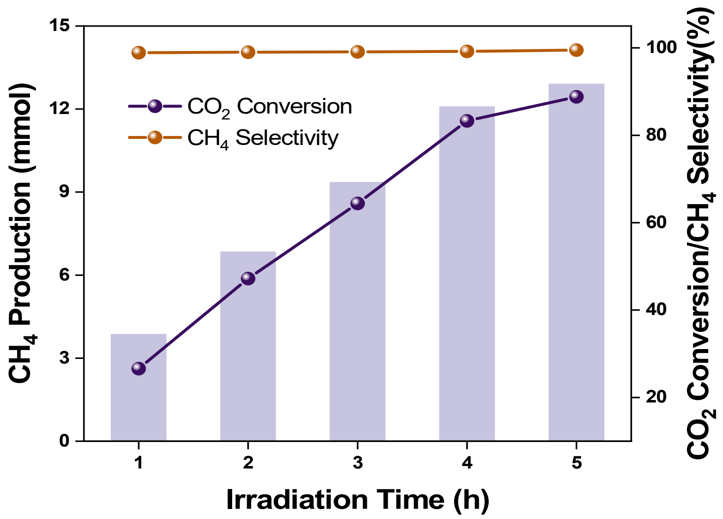

Supplementary Fig.20. Influence of irradiation time on CH_4 production over Co@NC-700 catalyst. Reaction conditions: 250 °C, initial pressure 10 bar (H_2/CO_2=4/1), 15 mg catalyst, full-arc 300 W UV-xenon lamp, 2.5 W cm^{-2}.

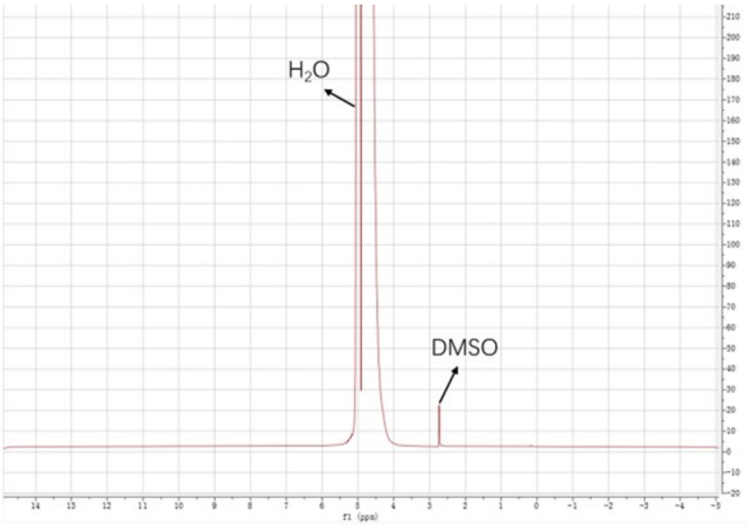

Supplementary Fig.21. The ^{1}H NMR spectroscopy of the liquid samples after the reaction (DMSO as internal standard).

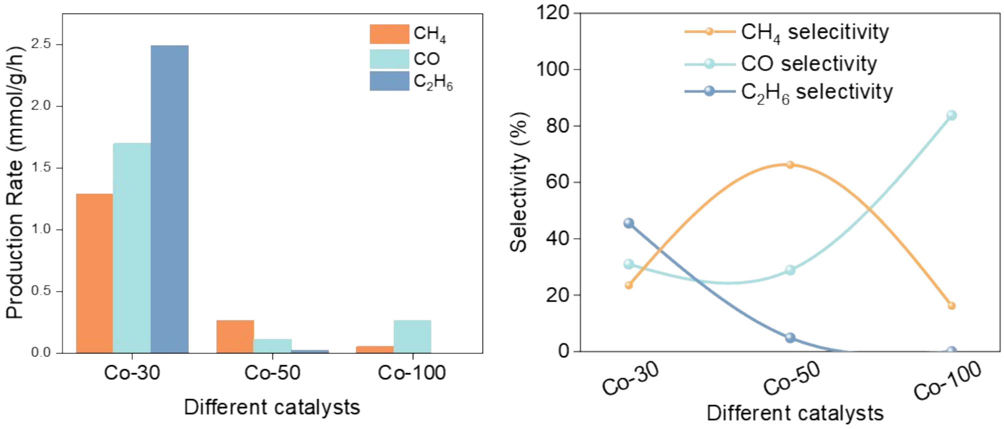

Supplementary Fig.22. CH_4 evolution rate and products selectivity over different catalysts.

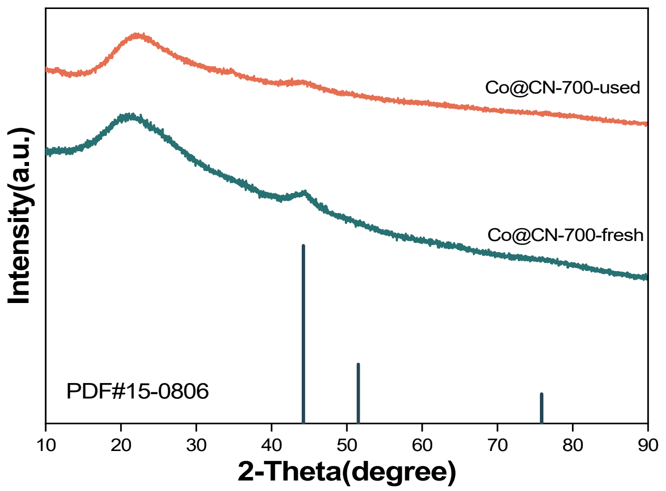

Supplementary Fig.23. XRD patterns of Co@NC-700 before and after 9 cycles of reaction.

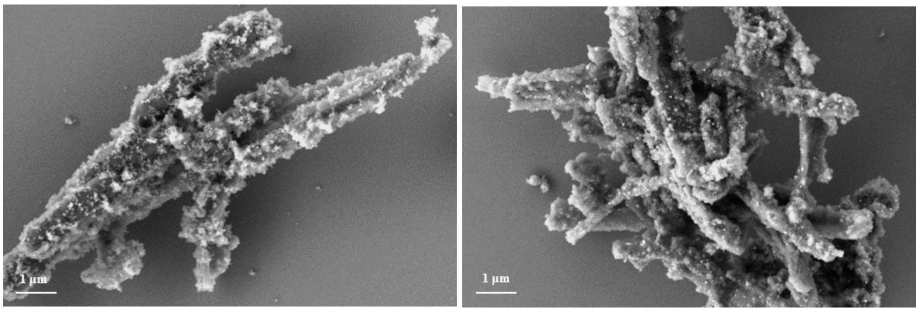

Supplementary Fig.24. SEM images of the used Co@CN-700 catalyst after 9 cycles of reaction.

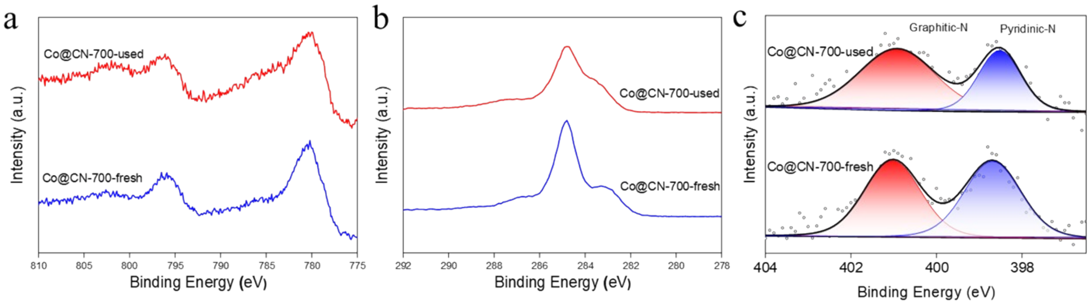

Supplementary Fig.25. High-resolution Co 2*p* (a), C 1*s* (b) and N 1*s* (c) XPS spectra of the fresh and used (9 cycles) Co@CN-700 catalysts.

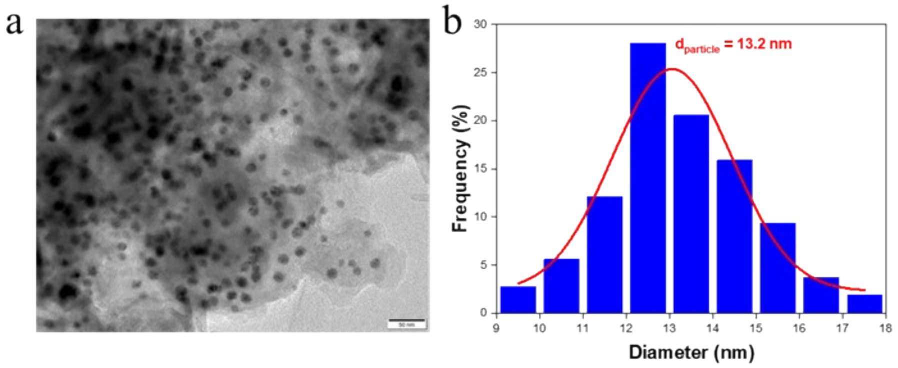

Supplementary Fig.26. The TEM photograph (a) and average distribution of Co NPs size (b) of Co@CN-700 sample after 9 cycles.

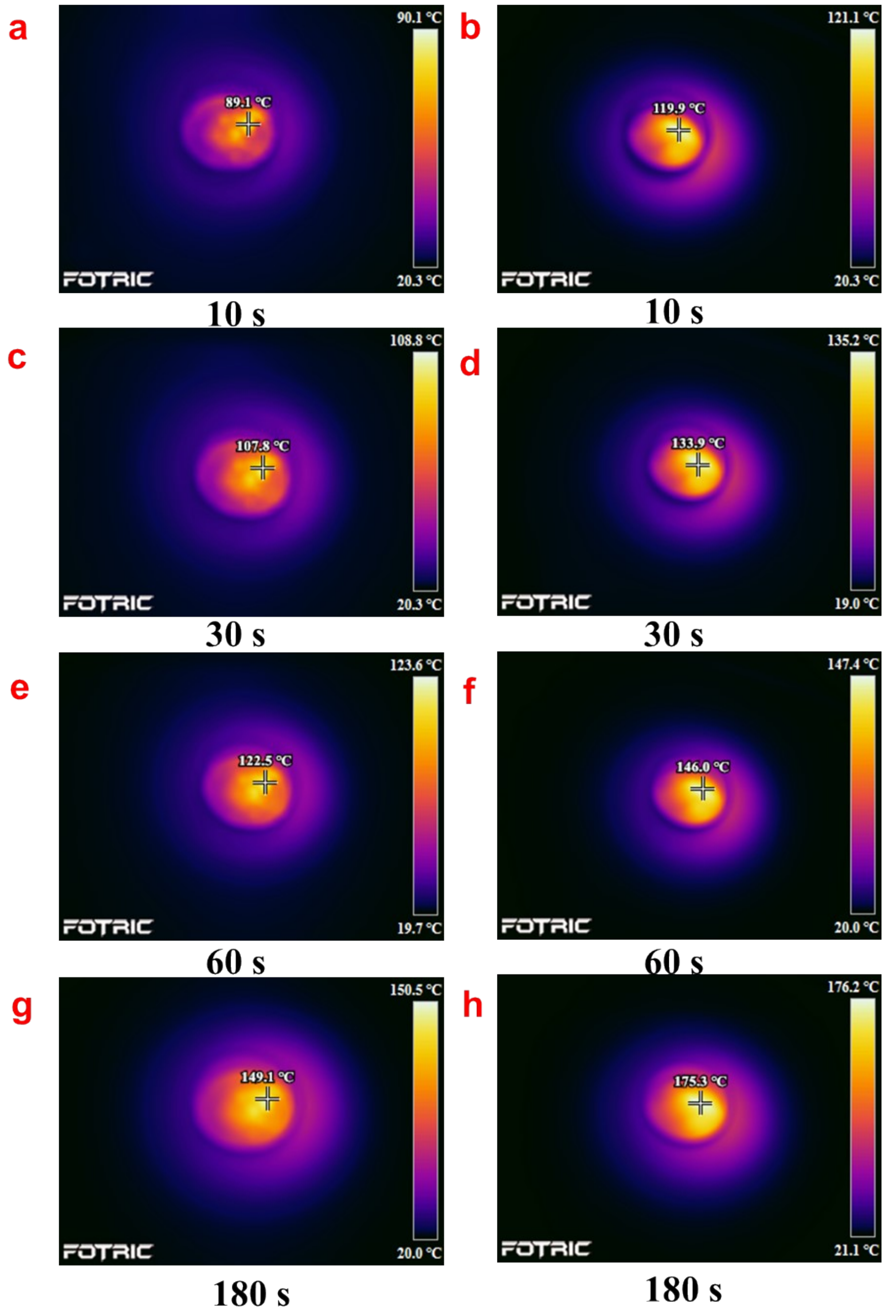

Supplementary Fig.27. Photothermal effect of different catalysts. Infrared temperature images after 10 s irradiation for CN-700 (**a**) and Co@CN-700(**b**). Infrared temperature images after 30 s irradiation for CN-700 (**c**) and Co@CN-700(**d**). Infrared temperature images after 60 s irradiation for CN-700 (**e**) and Co@CN-700(**f**). Infrared temperature images after 180 s irradiation for CN-700 (**g**) and Co@CN-700(**h**).

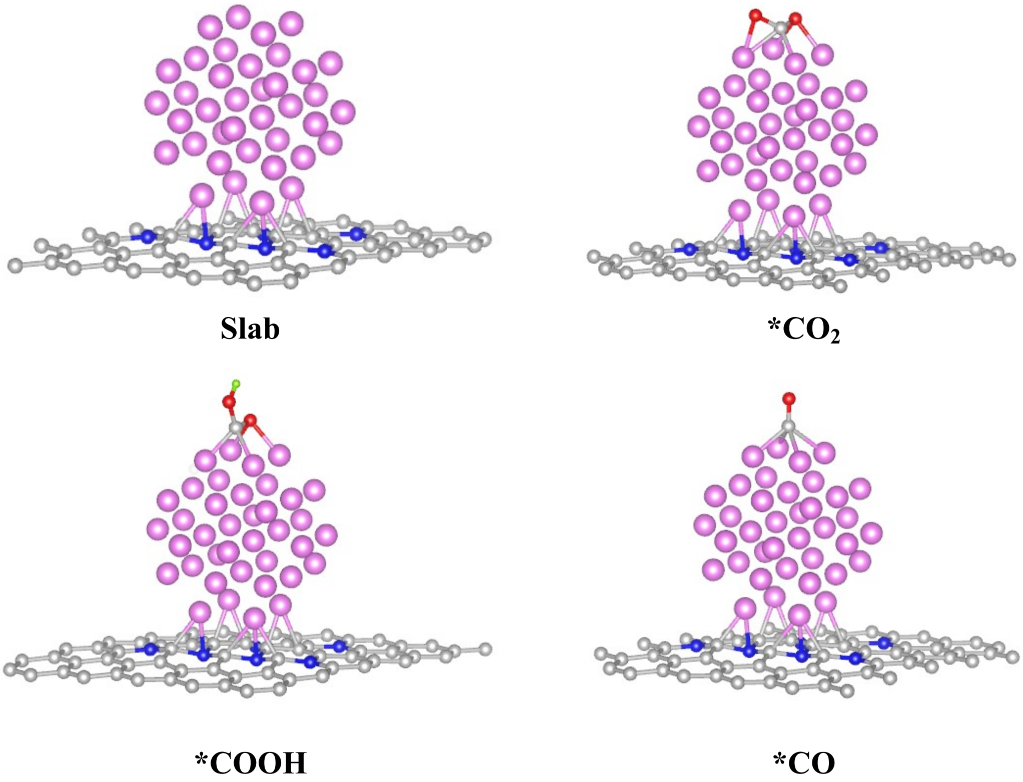

Supplementary Fig.28. Intermediates structures of CO_2 hydrogenation. C atoms (gray), N atoms (blue), O atoms (red), H atoms (green), and Co atoms (pink).

Supplementary Table S1. The summarized CH_4 yields for recently reported photo-thermo-catalysts. Comparison of the catalytic performance of non-noble catalysts for CO_2 methanation.

Catalysts	H_2:CO_2 ratio	Reactor	Light intensity (W cm^{-2})	Temperature (°C)	CH_4 production rate (mmol g_{cat}^{-1} h^{-1})	CO_2 conversion (%)	CH_4 selectivity (%)	Ref
Ni/TiO_2	4:1	flow	15.3	283	63.0	–	96.9	[7]
Ni/Nb_2C	1:1	batch	3.6	–	72.5	–	83.4	[8]
Ni/$BaTiO_3$	4:1	batch	0.293	270	103.7	38.0	100.0	[9]
Cu_2O/Graphene	4:1	batch	2	250 (External heating)	14.9 (Cu)	2.84	99	[10]
Co/Al_2O_3	4:1	batch	1.3	292	6.0	–	97.7	[11]
$Co_7Cu_1Mn_1O_x$	3:1	flow	0.234	200 (External heating)	14.5	27.45	85.3	[12]
CoFe-550	4:1	batch	5.2	310	1.7	68.2	90.9	[13]
Co_{10}/La_x-TiO_2	4:1	flow	0.5	450 (External heating)	6.1	30.5	83.2	[14]
Co_3O_4	4:1	batch	-	200 (External heating)	20.2	51.3	99.1	[15]
Ni/TiO_2	4:1	batch	2.5	250 (External heating)	15.3	-	-	[a]
Co@CN-700	4:1	batch	2.5	250 (External heating)	199.4	85.8	99	This work

[a] The Ni/TiO_2 was synthesized to text the catalytic performance in the batch system according to the ref. 7.

Supplementary Table S2. Physicochemical properties of various Co@CN-x catalysts.

Catalyst	Co loading(wt%)[a]	S_{BET}(m^2 g^{-1})	D_p(nm)
Co@CN-500	6.3	326.2	14.8
Co@CN-600	7.7	374.8	14.8
CN-700	-	428.6	14.8
Co@CN-700	8.3	397.2	14.9
Co@CN-800	11.3	412.3	14.9

[a] Determined by ICP-OES analysis.

Supplementary Table S3. Fitting parameters at the Co K-edge for various samples (S_0^2=0.85).

Sample	Shell	N^a	R(Å)^b	$\sigma^2\times10^3$(Å^2)^c	ΔE_0 (eV)^d	R factor
Co Foil	Co-Co	12	2.49±0.004	7.2±0.2	7.2±0.6	0.005
CoO	Co-O	4	2.12	1.7±2.3	-3.5±1.7	0.009
	Co-O	2	1.97	2.1±4.0		
Co@CN-600	Co-Co	4.2±0.9	2.50±0.02	4.2±1.8	-2.3±2.2	0.019
Co@CN-700	Co-Co	5.5±1.1	2.46±0.01	5.6±1.7	5.6±2.1	0.022
Co@CN-800	Co-Co	7.7±0.5	2.49±0.005	6.0±0.6	7.6±0.7	0.007

$^a N$: coordination numbers; $^b R$: bond distance; $^c\sigma^2$: Debye-Waller factors; ^{d} ΔE_0: the inner potential correction. R factor: goodness of fit.

References:

(1) Kresse, G.; Furthmüller, J. Efficiency of ab-initio total energy calculations for metals and semiconductors using a plane-wave basis set. *Comput. Mater. Sci.* **1996**, *6*, 15-50.

(2) Kresse, G.; Joubert, D. From ultrasoft pseudopotentials to the projector augmented-wave method. *Phys. Rev. B* **1999**, *59*, 1758-1775.

(3) Perdew, J. P.; Burke, K.; Ernzerhof, M. Generalized gradient approximation made simple. *Phys. Rev. Lett.* **1996**, *77*, 3865-3868.

(4) Grimme, S.; Antony, J.; Ehrlich, S.; Krieg, H. A consistent and accurate ab initio parametrization of density functional dispersion correction (DFT-D) for the 94 elements H-Pu. *J. Chem. Phys.* **2010**, *132,* 154104.

(5) Chadi, D. J. Special points for brillouin-zone integrations. *Phys. Rev. B* **1977**, *16*, 1746-1747.

(6) Henkelman, G.; Jónsson, H. Improved tangent estimate in the nudged elastic band method for finding minimum energy paths and saddle points. *J. Chem. Phys.* **2000**, *113*, 9978-9985.

(7) Li, Q.; Wang, C.; Wang, H.; Chen, J.; Chen, J.; Jia, H. Disclosing support-size-dependent effect on ambient light-driven photothermal CO_2 hydrogenation over nickel/titanium dioxide. *Angew. Chem., Int. Ed.* **2024**, *63*, e202318166.

(8) Wu, Z.; Li, C.; Li, Z.; Feng, K.; Cai, M.; Zhang, D.; Wang, S.; Chu, M.; Zhang, C.; Shen, J.; et al. Niobium and titanium carbides (Mxenes) as superior photothermal supports for CO_2 photocatalysis. *ACS Nano* **2021**, *15*, 5696-5705.

(9) Mateo, D.; Morlanes, N.; Maity, P.; Shterk, G.; Mohammed, O. F.; Gascon, J. Efficient visible-light driven photothermal conversion of CO_2 to methane by nickel nanoparticles supported on barium titanate. *Adv. Funct. Mater.* **2020**, *31,* 2008244.

(10) Mateo, D.; Albero, J.; García, H. Photoassisted methanation using Cu_2O nanoparticles supported on graphene as a photocatalyst. *Energy Environ. Sci.* **2017**, *10*, 2392-2400.

(11) Chen, X.; Li, Q.; Zhang, M.; Li, J.; Cai, S.; Chen, J.; Jia, H. MOF-templated preparation of highly dispersed Co/Al_2O_3 composite as the photothermal catalyst with high solar-to-fuel efficiency for CO_2 methanation. *ACS Appl. Mater. Interfaces* **2020**, *12*, 39304-39317.

(12) He, Z.-H.; Li, Z.-H.; Wang, Z.-Y.; Wang, K.; Sun, Y.-C.; Wang, S.-W.; Wang, W.-T.; Yang, Y.; Liu, Z.-T. Photothermal CO_2 hydrogenation to hydrocarbons over trimetallic Co–Cu–Mn catalysts. *Green Chem.* **2021**, *23*, 5775-5785.

(13) Chen, G.; Gao, R.; Zhao, Y.; Li, Z.; Waterhouse, G. I. N.; Shi, R.; Zhao, J.; Zhang, M.; Shang, L.; Sheng, G.; et al. Alumina-supported CoFe alloy catalysts derived from layered-double-hydroxide nanosheets for efficient photothermal CO_2 hydrogenation to hydrocarbons. *Adv. Mater.* **2017**, *30,* 1704663.

(14) Ullah, S.; Lovell, E. C.; Tan, T. H.; Xie, B.; Kumar, P. V.; Amal, R.; Scott, J. Photoenhanced CO_2 methanation over La_2O_3 promoted Co/TiO_2 catalysts. *Appl. Catal., B* **2021**, *294*, 120248.

(15) Gu, Y.; Ding, J.; Tong, X.; Yao, H.; Yang, R.; Zhong, Q. Photothermal catalyzed hydrogenation of carbon dioxide over porous nanosheet Co_3O_4. *J. CO_2 Util.* **2022**, *61*, 102003.
